# Preliminary results of synchronous acquired PET/MRI for viability assessment in patients with reduced cardiac function prior revascularization

**DOI:** 10.1186/1532-429X-17-S1-P108

**Published:** 2015-02-03

**Authors:** Christian F Luecke, Barbara Oppolzer, Peter Werner, Thies Jochimsen, Philipp Lurz, Matthias Grothoff, Henryk Barthel, Osama Sabri, Matthias Gutberlet

**Affiliations:** Department of Diagnostic and Interventional Radiology, University Leipzig - Heart Center, Leipzig, Germany; Nuclear Medicine Clinic, University Leipzig, Leipzig, Germany; Internal Medicine/Cardiology, University Leipzig - Heart Center, Leipzig, Germany

## Background

In the STICH trial viability assessed by stress-Echo or SPECT did not prove as a independent prognostic factor in patients prior revascularization. The question was raised if CMR and PET would have changed the results of the STICH trial. Simultaneous acquired PET/MRI has become recently available, combining the gold standards for viability assessment.

## Methods

We wanted to assess the interrater reliability of viability assessment in a hybrid PET/MR system, to assess if it could have additional value for patients prior revascularization. Therefore we examined 9 patients, scheduled for viability assessment by PET as a simultaneous PET/MR examination (mMR Biograph, Siemens, Erlangen, Germany). All patients were examined 50-60'after i.v. application of a mean activity of 319 MBq. The MRI protocol consisted of Cine SSFP sequences in 2 chamber, 4 chamber and short axis orientation (SA). For late enhancement assessment phase sensitive inversion recovery sequences (PSIR) in SA were acquired 10-15 minutes after administration of 0.15 mmol/kg body weight Gadobutrol (Gadovist, Bayer Healthcare, Berlin, Germany). 7 patients got 50g glucose and a individual dose of insulin, depending on blood glucose level. In 2 patients suffering from type 2 diabetes mellitus a standardized glucose clamp was performed. One patient got an additional Tc-99-Tetrofosmin myocardium SPECT at rest. The myocardium was segmented according to the 17-segment-model of the AHA. Viability was assessed in a binary format (PET: normal=0, diminished FDG uptake 1; MR: normal=0, late enhancement present=1) and a 25% stepwise analysis. Cohens Kappa analysis was performed.

## Results

**Figure 1:**
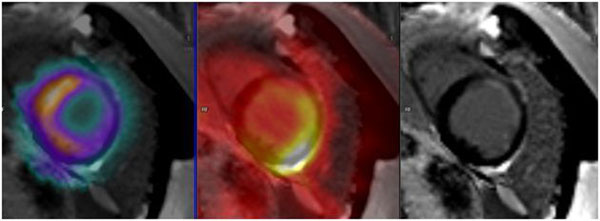
**(from right to left: Tc-99-SPECT, F-18-FDG-PET, PSIR-Late Enhancement)** While CMR shows subendocardial scar in the anterior and lateral segments, PET shows an intense glucose metabolism in the lateral wall. However the inferior and inferoseptal segments show low glucose metabolism although viable in MRI. An additionally performed TC-99-SPECT could exclude a perfusion deficit at rest, consistent with viable myocardium in the late enhancement sequence.

In total 153 segments were evaluated. The correlation between segments based on the binary assessment was substantial (k=0.645, z=12.3, p<0.05). However for the 25% step approach the correlation was poor (k=0.18, z=3.56, p< 0.05). Some segments did not show late enhancement but did also not show glucose uptake in F-18-FDG-PET. In one patient the additional Tc-99-Tetrofosmin myocardium SPECT could show persistent perfusion at rest, consistent with viable myocardium (Figure 1).

## Conclusions

Simultaneous viability assessment was feasible and did show a substantial agreement between methods in this small number of patients. Further analysis aims for an increase of the number of evaluated patients and the correlation of multimodal image data with the clinical outcome.

## Funding

This study was funded by the Heart Center Leipzig.

